# Oxidative Stress in Female Athletes Using Combined Oral Contraceptives

**DOI:** 10.1186/s40798-016-0064-x

**Published:** 2016-09-21

**Authors:** Sabina Cauci, Cinzia Buligan, Micaela Marangone, Maria Pia Francescato

**Affiliations:** Department of Medical and Biological Sciences, School of Medicine, University of Udine, Piazzale Kolbe 4, Udine, 33100 Italy

**Keywords:** Lipid peroxidation, Free radicals, Antioxidant defense, Physical activity, Contraceptive pill, Desogestrel, Drospirenone, Cyproterone, Hormonal contraception, Estrogen, Progestin, Third generation pill, Alimentary habits

## Abstract

**Background:**

Oxidative stress in female athletes is understudied. We investigated oxidative stress in sportswomen of different disciplines according to combined oral contraceptive (OC) use and lifestyle/alimentary habits.

**Methods:**

Italian sportswomen (*n* = 144; mean age 23.4 ± 4.2 years; body mass index 21.2 ± 2.2 kg m^−2^; sport activity 9.2 ± 4.1 h week^−1^) were analyzed; 48 % were volleyball players, 12.5 % soccer players, 10.4 % track-and-field sports, and followed by other disciplines’ athletes. Oxidative stress was evaluated by free oxygen radical test (FORT) assessing blood hydroperoxides and free oxygen radical defense (FORD) assay evaluating antioxidant capacity in OC users (*n* = 42) compared to non-OC users.

**Results:**

Elevated oxidative stress levels (≥310 FORT units) were found in 92.9 % of OC users and in 23.5 % of non-OC users (crude OR = 42, 95 % CI 12–149, *p* < 0.001; adjusted OR = 60, 95 % CI 11–322, *p* < 0.001). Continuous values of hydroperoxides were twofold higher in OC users versus non-OC users (median 484 versus 270 FORT units, *p* < 0.001) and were inversely related to FORD units in OC users (*p* = 0.01). Hydroperoxides were not associated with weekly hours of exercise. In OC users, lifestyle/alimentary habits were not correlated to hydroperoxides. In non-OC users only, hydroperoxide values were positively correlated with weight and BMI and inversely correlated with chocolate and fish consumption.

**Conclusions:**

The markedly elevated oxidative stress we revealed in OC-user athletes could be detrimental to physical activity and elevate cardiovascular risk (as thromboembolism). Further research is needed to extend our results, to clarify the biochemical pathways leading to increased hydroperoxides (mainly lipid peroxides) and reduced antioxidant defense, and to elucidate the potential effects on athletic performance. OC use should be considered when developing gender-focused strategies against oxidative stress.

## Key Points

- In female athletes of different disciplines, the use of combined oral contraceptive (OC) pills markedly elevated oxidative stress as evaluated by blood hydroperoxide levels.
- Oral contraceptives’ use seems to overwhelm the beneficial effects of some antioxidant food. Alimentary habits including chocolate and fish consumption appear to reduce oxidative stress only in female athletes not using OCs.
- Elevated oxidative stress in OC users could increase the cardiovascular risk of sportswomen and negatively affect athletic performance.

## Background

Oxidative stress is caused by an imbalance between the overproduction of reactive oxygen (ROS) or nitrogen (NOS) species (collectively called RONS) and the body detoxification mechanisms including scavenging molecules that orchestrate the antioxidant defense [1–4]. Free radicals are deleterious to the human body, because they can retrieve electrons from various molecules provoking formation of oxidized forms, so that, severe oxidative stress can even trigger cell apoptosis and necrosis [2]. Mounting evidence suggests that oxidative stress could play a pivotal role in the pathogenesis of several diseases including inflammatory, muscular, cardiovascular, and neurodegenerative diseases [2–5]. In the early 1980s, a pioneer study by Davies et al. [6] proposed the exercise-induced production of RONS. Presently, a great body of evidence suggests that RONS have several roles in exercise beyond the detrimental damaging effects; indeed, they participate in signaling to regulate muscle function and in initiating adaptive responses to exercise [4, 7, 8]. The increased production of free radicals and oxidative stress in the context of physical exercise, sport performance, and muscle function has largely been explored [1, 3, 5, 9, 10]. In athletes, oxidative stress is a recognized causative agent of muscle redox imbalance that can promote muscle fatigue and injury, thus impairing exercise performance [1, 3, 7, 9]. However, so far, a limited number of studies have focused on oxidative stress in female athletes [11–14]. A report [15] demonstrated that female athletes were more susceptible to oxidative stress than male athletes; however, this study did not evaluate contraception, a major issue to be addressed in female athletes [16]. It is known that female athletes use OCs not only exclusive for birth control but also to regulate menses, to possibly plan competitive events, and to prevent premenstrual/menstrual or endometriosis pain [17]. The serum effects of OC use in athletes are minimally studied [18], although some evidence of decreased performance is emerging [19]. Surprisingly, only one study investigated oxidative stress in sportswomen according to OC use reporting significantly higher resting oxidative stress in 12 female judoist OC users than in 14 non-OC-user judoists [20]. Moreover, few studies assessed oxidative stress in OC-user women of the general population [21–25].

Progress in the understanding of mechanisms inducing oxidative stress in female athletes can provide important insights into the complex pathophysiology of oxidative stress-induced damages and could open the way to forthcoming studies also regarding the antioxidant requirements of sportswomen.

We aimed to analyze the oxidative stress levels in young female athletes according to OC use also taking into account lifestyles and alimentary habits of study participants. Specifically, we evaluated the effects of OC use on continuous and stratified elevated hydroperoxide levels, to contribute understanding of hormonal treatment effects in sportswomen.

## Methods

### Population

Study participants were enrolled consecutively for an observational study approved by the Local Institutional Ethical Committee, in accord with the Declaration of Helsinki. All female athletes signed a written informed consent before entering the study.

Healthy white Italian women (age range 18–45 years) regularly performing five or more hours weekly of sport activity, including training and competitions, during the competitive season were recruited as volunteers for study participation (including capillary blood donation and a questionnaire filling out). Enrollment was done through announcements at the Sport Sciences Campus in Gemona of Udine University. Before entering the study, each female athlete was interviewed to assess the inclusion criteria: (a) absence of any acute (current/recent infection) or chronic disease (thyroid or autoimmune diseases, celiac disease, diabetes mellitus, cardiovascular disease, or tumor etc.); (b) no recent muscle injury/pain or current pain from whatever cause; (c) no use of anabolic or other doping substance; (d) no hormonal treatment other than monophasic combined contraceptive pills [18, 26]; (e) not lactating, pregnant, or postmenopausal [27, 28]; (f) no present or past menstrual dysfunction (such as amenorrhea and menorrhagia) [18, 29]; and (g) no major disorders of sleeping (such as jet lag or sleeping less than 6 h the night before blood testing). Eligible women for inclusion in the case group were those using a monophasic combined oral contraceptive for at least 3 months [18, 26]. For inclusion in the control group women had never used hormonal contraception or had discontinued any hormonal treatment for more than 3 months [18, 26]. Out of 150 female athletes screened, 144 participants were eligible for the study of which 42 were OC users and 102 were non-OC users. Each participant completed a self-administered questionnaire assessing demographic factors, medical history, and lifestyle habits (including smoking). In addition, athletes filled a 2-week long diary of alimentary habits expressed as daily number of servings as described in Table 1. Regarding the use of alimentary supplements, athletes consumed a wide variety of different commercial products including salts, vitamins, minerals (especially iron and zinc), amino acids, protein products, and algae as single or mixed formulations. Some athletes were using more than one product. None of the women were taking vitamin C alone as supplement; however, vitamin C was included in several multi-substance supplement formulations. Moreover, supplement dose was variable in quantity and consistency of assumption (occasional, intermittent, and continual). Consequently, we did not categorize alimentary supplements into specific subgroups, and we used supplement use as a categorical variable, yes or no, for logistic regression analysis. For statistical reporting of data, average numbers of servings were reported as mean ± standard deviation (SD) of weekly or daily use as indicated. Body mass index (BMI) was calculated as weight (kg) divided by the square of height (m).

**Table 1:** Demographic, lifestyle, alimentary characteristics, and oxidative stress (by FORT units) of 144 study sportswomen, comparison between the 42 OC users and 102 non-OC users

Characteristic	All athletes (*n* = 144) mean ± SD or *n* (%)	OC users (*n* = 42) mean ± SD or *n* (%)	Non-OC users (*n* = 102) mean ± SD or *n* (%)	*p*
Sport activity, hours week^−1^	9.2 ± 4.15	9.3 ± 5.60	9.1 ± 3.40	0.64^b^
Elite athletes (national/international), *n* (%)	27 (18.7)	7 (16.7)	20 (19.6)	0.68^c^
Age, years	23.4 ± 4.17	23.4 ± 3.62	23.4 ± 4.40	0.67^b^
Weight, kg	61.5 ± 8.69	61.2 ± 7.79	61.6 ± 9.07	0.86^b^
Height, cm	170 ± 6.81	170 ± 6.97	170 ± 6.76	0.76^b^
BMI, kg m^−2^	21.2 ± 2.20	21.2 ± 2.02	21.1 ± 2.28	0.78^b^
University education, *n* (%)	104 (72.2)	34 (81.0)	70 (68.6)	0.13^c^
Married or separated/divorced, *n* (%)	12 (8.3)	1 (2.4)	11 (10.8)	0.18^d^
Nulliparity, *n* (%)	143 (99.3)	42 (100)	101 (99.0)	1.00^d^
Smokers, *n* (%)	33 (22.9)	14 (33.3)	19 (18.6)	0.06^c^
Cigarettes, day^−1^	1.1 ± 3.05	1.4 ± 2.41	1.0 ± 3.28	0.06^b^
Coffee drinkers, *n* (%)	116 (80.6)	32 (76.1)	84 (82.4)	0.40^c^
Coffee, cups day^−1^	1.5 ± 1.14	1.3 ± 1.12	1.6 ± 1.15	0.13^b^
Tea drinkers, *n* (%)	104 (72.2)	34 (81.0)	70 (68.6)	0.13^c^
Tea, cups day^−1^	0.5 ± 0.65	0.7 ± 0.62	0.4 ± 0.52	0.018^b^
Milk, cups week^−1^	4.1 ± 3.68	3.0 ± 2.98	4.5 ± 3.86	0.012^b^
Chocolate, 50 g servings week^−1^	2.0 ± 1.91	1.3 ± 1.35	2.3 ± 2.05	0.010^b^
Bread, 50 g servings week^−1^	7.0 ± 3.51	6.8 ± 3.85	7.0 ± 3.36	0.62^b^
Rice, 80 g servings week^−1^	1.2 ± 1.05	1.2 ± 0.86	1.2 ± 1.13	0.24^b^
Pasta, 100 g servings week^−1^	3.8 ± 1.80	3.6 ± 1.50	3.9 ± 1.92	0.55^b^
Fruits, 200 g servings week^−1^	7.0 ± 5.36	6.4 ± 3.91	7.2 ± 5.87	0.87^b^
Tomato/eggplant/pepper, plates week^−1^	2.2 ± 2.08	2.6 ± 1.99	2.1 ± 2.12	0.12^b^
Fresh vegetables, plates week^−1^	7.9 ± 4.23	7.7 ± 4.22	8.1 ± 4.25	0.35^b^
Legumes, plates week^−1^	0.7 ± 0.84	0.9 ± 1.11	0.6 ± 0.67	0.17^b^
Cheese, 50 g servings week^−1^	2.8 ± 2.06	2.5 ± 1.79	2.9 ± 2.16	0.45^b^
Eggs, number week^−1^	0.9 ± 0.70	0.8 ± 0.73	0.9 ± 0.69	0.24^b^
Red meat, 150 g servings week^−1^	1.7 ± 1.14	1.7 ± 1.07	1.7 ± 1.18	0.82^b^
Total meat (any type), 150 g servings week^−1^	4.0 ± 1.75	3.7 ± 1.62	4.2 ± 1.80	0.26^b^
Sausages, 50 g servings week^−1^	1.82 ± 1.42	1.7 ± 1.09	1.9 ± 1.54	0.77^b^
Fish, 200 g servings week^−1^	1.1 ± 0.86	1.1 ± 0.87	1.1 ± 0.86	0.97^b^
Yogurt, 125 g servings week^−1^	2.0 ± 2.10	2.1 ± 2.12	2.0 ± 2.11	0.64^b^
Sweet cakes, 50 g servings week^−1^	5.2 ± 3.48	4.6 ± 3.74	5.5 ± 3.34	0.11^b^
Wine, 125 mL glasses week^−1^	0.8 ± 1.38	1.0 ± 1.46	0.8 ± 1.35	0.46^b^
Beer, 200 mL glasses week^−1^	1.4 ± 2.18	2.1 ± 3.13	1.1 ± 1.53	0.10^b^
Spirits, 40 mL glasses week^−1^	0.3 ± 0.57	0.3 ± 0.46	0.3 ± 0.61	0.62^b^
Total alcohol grams week^−1^	40 ± 49.9	52 ± 67.2	35 ± 39.6	0.25^b^
Supplement use, *n* (%)	34 (23.6)	14 (33.3)	20 (19.6)	0.08^c^

*BMI* body mass index

^a^Italian espresso coffee cups

^b^ *p* comparison of OC users and non-OC users by two-tailed Mann-Whitney test

^c^ *p* comparison of OC users and non-OC users by two-tailed Pearson’s chi-squared test, as appropriate

^d^ *p* comparison of OC users and non-OC users by two-tailed Fisher’s exact test, as appropriate

### Oxidative Stress Evaluation

All participants were asked to refrain from physical exercise, alcohol, and supplement consumption 24 h before blood retrieval [18, 29]. All women were enrolled randomly with respect to menstrual cycle, but menstruation bleeding days were avoided (days 1–7 of menstrual cycle), because contraceptive pill dosage consists of discontinuation of drug or placebo use in these days. Finger capillary blood samples were obtained in duplicate (within 20 min apart) for each measured parameter from seated and 12 h fasting subjects in the morning (between 8 and 10 am) [30]. Personnel performing the collection and measurement of samples were blinded to clinical, demographic, and habit data. Reactive oxygen species, in the form of hydroperoxides (considered to represent blood lipid hydroperoxides [30]), were determined as previously described [30] in 20 μL of capillary blood using the free oxygen radical test (FORT assay; Callegari, Parma, Italy), a 6-min long colorimetric assay based on the ability of transition metals to catalyze the breakdown of hydroperoxides (ROOH) into radicals, according to the Fenton reaction. Results were expressed as FORT units, whereby 1 FORT unit corresponded to 0.26 mg/L H_2_O_2_ [30]. The intra- and inter-assay variations were <4.5 and <5.0 %, respectively. Detection limits of the assay were ≤160 and ≥600 FORT units. Stratification of hydroperoxides was performed by the threshold of 310 FORT units as high level, and of 400 FORT units attesting very high levels of oxidative stress [31].

Two further capillary samples (50 μL each) were drawn [30] to determine the free oxygen radical defense (FORD assay; Callegari, Parma, Italy) evaluating the blood total antioxidant capacity comprising ascorbic acid, glutathione, and albumin (but not uric acid) [32]. The total time for the four capillary blood samples retrieval was less than 1 h. Due to poor compliance of athletes to the third and fourth retrieval; the FORD assay was performed in all the 42 OC users and in randomly selected consecutively consenting 42 non-OC users. We decided to use the FORT and FORD assays because, beside avoiding invasive venous blood retrieval, these point of care assays have been recently validated for use in athletes and might be used easily in sport medicine facilities [32]. The individual biological variations of FORT and FORD assays were determined by Lewis and colleagues [32] as 5.0 and 7.5 %, respectively.

### Statistical Analysis

The Kolmogorov-Smirnov test was used to assess normality of distribution of variables. Normally distributed data were presented as mean ± SD. The FORT and FORD data resulted skewed, thus, median and interquartile (IQR, 25th to 75th percentile) values were reported and non-parametric tests used. The Mann-Whitney *U* test was used for comparison of continuous variables. The difference of proportions between OC users and non-OC users was assessed by Pearson’s *χ*^2^-test or Fisher’s exact test, as appropriate; specifically, Fisher’s test was used for variables “married or separated/divorced” and “nulliparity”; for all other categorical variables, Pearson’s test was appropriate. Crude odds ratios (ORs) and 95 % confidence intervals (CIs) were evaluated for categorical variables. At an alpha level of 0.05, we had 90 % power to detect a difference in hydroperoxides between the OC and the non-OC groups. Logistic regression was performed to evaluate the difference in oxidative stress between groups after adjustment for age, BMI, smoking and supplement use, tea, chocolate, fresh vegetable, and fish servings per week. Bivariate relationships were evaluated by the Spearman rho test (*r*_*s*_). All tests were two-sided. *p* values <0.05 were considered statistically significant, *p* < 0.10 values were considered a tendency. Statistical analysis was performed using the Statistical Package for Social Sciences (SPSS for Windows, SPSS Inc., Chicago, IL, USA).

## Results

### Sport Activities

Nineteen percent (27/144) of participants were elite athletes with national and/or international competitive experience; however, the majority of athletes (117/144, 81.2 %) were non-elite grade competing at regional levels (mostly in Friuli-Venezia-Giulia Region, Northern Italy). Approximately half of the athletes (47.9 %) were volleyball players, followed by soccer players (12.5 %), track-and-field athletes (10.4 %), runners (6.9 %), figure skating/artistic gymnastics/competitive dancing athletes (6.2 %), basketball players (5.6 %), martial arts athletes (3.5 %), swimmers (2.1 %), skiers (2.1 %), cyclists (1.4 %), and weight lifters (1.4 %).

### Combined Oral Contraception

Twenty-nine percent (42/144) of the athletes were using OC from 33 ± 32.6 months (3–140 months range). OCs were all monophasic combined pills but had different formulations, with ethinyl-estrogenic component ranging from 15 to 35 μg and variable progestin components: approximately half preparations contained gestodene (45.2 %, 19/42), followed by drospirenone (21.4 %, 9/42), desogestrel (14.3 %, 6/42), cyproterone (9.5 %, 4/42), levonorgestrel (7.1 %, 3/42), and clormadinone (2.4 %, 1/42). Overall, third generation pill preparations containing either gestodene or desogestrel were used by 59.5 % (25/42) of OC users. Preparations containing progestins with the highest risk of venous thromboembolism according to recent evidence [33] including desogestrel, cyproterone, and drospirenone were used by 45.2 % (19/42) of OC users.

### Characteristics and Alimentary Habits of the Study Female Athletes

The main demographic characteristics, lifestyle, and alimentary habits of the 144 female athletes are described in Table 1. Participants performed on average 9.2 ± 4.15 h weekly of regular physical activity including training and competitions, they were 23.4 ± 4.17 years old, most had university level education, were mostly unmarried and nulliparous, all had a middle-class socioeconomic status, and 22.9 % were smokers. The majority of women (92.3 %) were in the normal weight range (BMI ≥18.0 and ≤25.0 kg m^−2^), five were underweight women (3.5 %), and six overweight women (4.2 %); however, none of the women were obese (i.e., BMI ≥30.0 kg m^−2^). As illustrated in Table 1, OC users were not different from non-OC users with regard to the majority of the studied parameters. However, the OC users had more cups of tea (*p* = 0.018), fewer cups of milk (*p* = 0.012), and fewer chocolate servings (*p* = 0.010) than non-OC users.

**Fig. 1:**
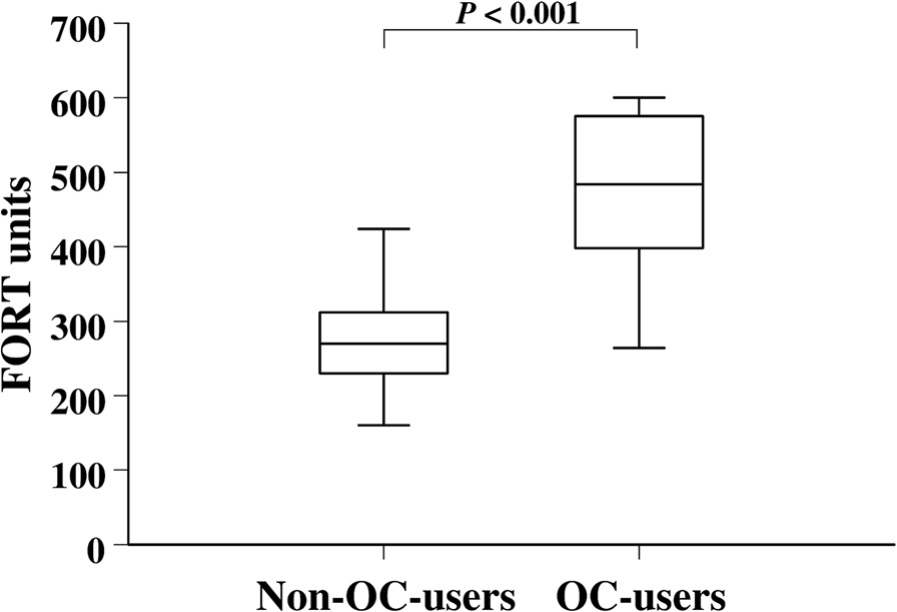
Hydroperoxide levels in female athletes according to OC use. Box and whiskers plots comparing the hydroperoxide values (in FORT units) in non-OC users and OC-user female athletes, respectively. *Horizontal line* is the median, *box* represents interquartile, and *whiskers* represent extreme values

Continuous values of hydroperoxides were almost twofold higher in OC users compared to non-OC users, median 484 (IQR, 397–581) versus median 270 (IQR, 230–312) FORT units, *p* < 0.001, as shown in Fig. 1. Notably, high values of hydroperoxides ≥310 FORT units were found in 92.9 % (39/42) of OC users versus 23.5 % (24/102) of non-OC users, crude OR = 42, 95 % CI 12–149, *p* < 0.001. The OR remained highly significant as well after multivariate analysis adjusting for age, BMI, smoking and supplement use, tea, chocolate, fresh vegetable, and fish servings per week, adjusted OR = 60, 95 % CI 11–322, *p* < 0.001. Very high levels of hydroperoxides ≥400 FORT units were observed in 71.4 % (30/42) of OC users versus 1.0 % (1/102) of non-OC users, crude OR = 252, 95 % CI 32–2022, *p* < 0.001, adjusted OR = 315, 95 % CI 31–3145, *p* < 0.001.

Supplement users compared to non-users did not differ in frequency of elevated hydroperoxides (≥310 FORT units) in the entire cohort, in OC users, and in non-OC-user groups.

Continuous values of free oxygen radical defense capacity in FORD units were lower in 42 OC users compared to 42 non-OC users, median 0.90 (IQR, 0.82–1.06) versus median 1.08 (IQR, 1.00–1.18) FORD units, *p* = 0.001.

By comparison of FORT and FORD continuous values among the 19 OC users of OCs containing desogestrel, cyproterone, and drospirenone versus the 23 users of OCs with different progestins, FORT and FORD units values did not differ significantly between the two subgroups, although FORT continuous values had a tendency to be higher (median 518 versus median 446, *p* = 0.076) in the former subgroup.

By comparison of FORT and FORD continuous values in smokers versus non-smokers among OC users and among non-OC users, no significant differences were found.

By comparison of FORT and FORD continuous values in supplement users versus non-users among OC users and among non-OC users, no significant differences were found.

### Correlations of Oxidative Stress According to OC Use

Table 2 shows correlations of hydroperoxide continuous values (in FORT units) and characteristics of female athletes according to OC use. Hydroperoxide values were not associated with hours of physical exercise per week in OC users and in non-OC users.

**Table 2:** Relationships of continuous lipid peroxidation values (in FORT units) with other parameters by two-tailed Spearman correlation coefficient (*r*_*s*_) in the 42 OC users and 102 non-OC users

Characteristic	OC users (*n* = 42)	Non-OC users (*n* = 102)
Sport activity, hours week^−1^	−0.18	0.11
Age, years	−0.10	0.06
Weight, kg	−0.19	0.26**
Height, cm	−0.26	0.02
BMI, kg m^−2^	0.01	0.30**
Time of OC use, months	0.03	−
Cigarettes, day^−1^	−0.19	−0.09
Coffee, cups^a^ day^−1^	−0.25	−0.10
Tea, cups day^−1^	0.16	0.16
Milk, cups week^−1^	0.07	0.10
Chocolate, 50 g servings week^−1^	−0.11	−0.29**
Bread, 50 g servings week^−1^	−0.19	−0.16
Rice, 80 g servings week^−1^	0.06	−0.14
Pasta, 100 g servings week^−1^	0.02	0.03
Fruits, 200 g servings week^−1^	−0.19	−0.10
Tomato/eggplant/pepper, plates week^−1^	−0.04	0.01
Fresh vegetables, plates week^−1^	−0.28^	0.02
Legumes, plates week^−1^	−0.04	0.07
Cheese, 50 g servings week^−1^	−0.11	−0.10
Eggs, servings week^−1^	−0.12	0.13
Red meat, 150 g servings week^−1^	−0.01	−0.17
Total meat (any type), 150 g servings week^−1^	0.15	−0.13
Sausages, 50 g servings week^−1^	−0.08	0.01
Fish, 200 g servings week^−1^	−0.18	−0.22*
Yogurt, 125 g servings week^−1^	−0.02	−0.12
Sweet cakes, 50 g servings week^−1^	−0.14	0.07
Wine, 125 mL glasses week^−1^	0.09	0.01
Beer, 200 mL glasses week^−1^	0.17	0.08
Spirits, 40 mL glasses week^−1^	−0.05	0.11
Total alcohol, grams week^−1^	0.09	0.13

Significant findings are indicated in italics. Superscript “^” indicated a tendency

*BMI* body mass index

*p < 0.05; **p < 0.01

^a^Italian espresso coffee cups

In OC users, oxidative stress was not correlated to months of OC use, and no significant findings were observed regarding alimentary habits; however, fresh vegetable servings per week had a tendency for a negative correlation with hydroperoxides (*p* = 0.086). Interestingly, we found a positive association of oxidative stress with body weight (*p* = 0.009), and BMI (*p* = 0.002) in the 102 non-OC users only. Moreover, in non-OC users only, chocolate (*p* = 0.009) and fish (*p* = 0.029) number of servings per week were inversely related to hydroperoxides. No other significant finding was observed in relation to alimentary and smoking habits in non-OC users.

FORT units were inversely related to FORD units in the total 84 women examined (*r*_*s*_ = −0.60, *p* < 0.001), but by group analysis this relationship was statistically significant only in the 42 OC users (*r*_*s*_ = −0.54, *p* = 0.01), not in the 42 non-OC users.

## Discussion

Currently, the scientific interest in the effects of sport on oxidative stress levels is increasing both for health and performance implications [1, 3]. Several authors emphasize that additional research will be required in this field [1, 3] because discrepancies exist among studies especially regarding antioxidant strategies. Antioxidant and pro-oxidant pathophysiological pathways of physical activity need to be elucidated, apparently, beneficial effects on oxidative stress and health are obtained when regular moderate training is practiced [3]. On the contrary, acute exercise seems to increase oxidative stress, but intriguingly the oxidative stimulus is necessary to upregulate endogenous antioxidant defenses [7]. Gender-specific studies are warranted to take into account sex differences in factors potentially modulating oxidative stress [1, 34, 35].

Our observational study was the first to explore continuous and categorized oxidative stress levels according to OC use in a sample of 144 ethnically homogeneous white young adult female athletes practicing various sport disciplines. We found that oxidative stress levels (evaluated by hydroperoxides) vary considerably according to OC use, the median value of FORT units was almost twofold higher in OC users than non-OC users athletes. Moreover, by evaluating stratified levels of hydroperoxides, we observed markedly elevated frequencies in OC users compared to non-OC users with crude OR = 42, and adjusted OR = 60 for the ≥310 FORT units threshold, and dramatically elevated crude OR = 252, and adjusted OR = 315 considering the ≥400 FORT units threshold. Our findings highlighted a remarkable increase of elevated oxidative stress in OC-user athletes that was apparently not associated with their lifestyles and alimentary habits; likely, in OC users, antioxidant foods’ effects could be overwhelmed by pro-oxidant effects of OCs. Conversely, in non-OC users, we found negative relationships of oxidative stress with some alimentary habits, specifically, chocolate and fish servings per week. Our findings are consistent with the known antioxidant properties of chocolate/cocoa [36] and fish (rich in n3-polyunsaturated fatty acids) [37]. It is to mention that in our study, OC users had fewer chocolate servings per week than non-OC users, thus, the chocolate/cocoa effects will need further investigations.

In our study, we did not observe smoking and supplement use effects on oxidative stress levels in OC users and non-OC users. It is to be noted, however, that number of cigarettes smoked was very low (on average one cigarette per day) and that we asked participants to refrain from the use of supplements in the 24 h before blood testing; thus, it is very likely that some substances as vitamin C were washed-out. Particular effects of supplements will require specific further research.

In non-OC-user athletes only, our study highlighted a positive association between the FORT levels and BMI. Other authors found an association between BMI and systemic oxidative stress markers in the general population [38] and in active adults [39]. Of note, in our study, none of the athletes were obese; thus, we demonstrated that even in normal weight subjects oxidative stress had a positive association with increased BMI, likely representing progressive elevation of accumulated fat. However, we did not observe a relationship between oxidative stress and BMI in OC users. It seems plausible that the induction of oxidative stress by OC use can overshadow the effects related to BMI. Further investigations are warranted to assess this issue.

In OC users, we found an inverse relationship of hydroperoxides (FORT units) with total defense capacity against free oxygen radicals (FORD units), that did not reach significant values in non-OC users. However, our data do not permit to infer causality, i.e., the present study cannot assess whether OC use directly increased ROS production that provoked formation of hydroperoxides and consumed antioxidant defenses, and/or whether OC use directly reduced antioxidant defenses, which became insufficient to neutralize free radicals, in turn provoking hydroperoxidation.

Our current findings are in line with the only previous study examining oxidative stress (by assessment of malondialdehyde and lipid peroxides) in female athletes taking OC [20]. This study compared 12 female judoists using OC containing drospirenone and ethinylestradiol with 14 non-OC users, and found that OC users had significantly higher lipid peroxidation and lower antioxidant defense [20]. However, given the small number of subjects, the study did not perform multivariate analysis. Another study in non-athletic women found increased lipid peroxides (+176 %, *p* < 0.001) and oxidized low-density lipoproteins (LDLs) (+145 %, *p* < 0.002) in 32 OC users compared with 30 non-OC users [22]. A study in Belgian women aged 40–48 years found a significant increase of lipid peroxides in 209 OC users compared to 119 non-users of contraception [21]. An interesting study investigating time-course of hydroperoxide elevation in women users of a low estrogen dose pill containing drospirenone demonstrated that oxidative stress increased significantly after only 1 week of OC use, remained constantly elevated during OC use, and returned to basal levels within 1 week of OC discontinuation [24]. These results by Finco and colleagues [24] seem in line with our observation that hydroperoxides in OC users are not related to months of OC use.

Mechanisms leading to elevation of hydroperoxides by OC use still need to be clarified [24]. It is plausible that catabolism of exogenous hormones by involving activities of P450 cytochromes (CYPs) provokes increased ROS production [40] and depletion of reduced glutathione [24, 25]. Future studies are warranted to assess if the observed blood rise of oxidative stress associated with OC use is estrogens and/or progestin related [22, 24, 41]. Interestingly, a recent study showed that in vitro estradiol treatment of cells resulted in a significant increase in lipid peroxidation [42]. Contradictory findings were presented by other studies on pro- or antioxidant action of estrogens [22, 24, 43, 44]. Some evidence suggested that estrogens are inversely related to antioxidant defense, in particular, high estrogen levels were correlated to decreased blood superoxide dismutase (SOD) levels [44], but on the other hand, a study on female rats reported no relation of administered estrogen with SOD, but a positive relation with increased lipid peroxidation [43].

Further studies with increased number of female athletes will be necessary to evaluate the biochemical pathways of oxidative stress elevation in OC users.

New generations of OC pills are characterized by lower estrogen content and by newer progestins, like desogestrel, gestodene, cyproterone, and drospirenone, with lower androgenicity than past generation pills [41]. They have been introduced to reduce severe adverse effects of OC use, especially venous thromboembolism, and other cardiovascular diseases [17, 45]. However, these new OC preparations are still associated with the risk of myocardial infarction, thrombotic stroke, and venous thromboembolism [41, 46, 47]. The risk of venous thromboembolism associated with OC use is of particular concern and has been recently investigated in a total of 10,562 cases of thromboembolism by Vinogradova and colleagues [33]; exposure to OC containing desogestrel had increased risk OR of 4.28, cyproterone 4.27, drospirenone 4.12, gestodene 3.64, norethisterone 2.56, norgestimate 2.53, and levonorgestrel 2.38 in respect to no exposure to OCs in the previous year [33]. In our study, we did not find statically significant differences in FORT and FORD units between users of OC containing desogestrel, cyproterone, and drospirenone versus other progestins; however, we observed a tendency to higher hydroperoxide values in the first group of OC users. Larger studies will be necessary to assess this relevant issue.

The combined oral contraceptive pill is appreciated by sportswomen not only for birth control efficacy but also because OC provides a consistent 28-day cycle eliminating cycle-length variability and menstrual irregularities [48, 49]. Data on OC use and exercise capacity are sparse [48]. Some studies show that athletes on OCs experience a slight reduction in maximal aerobic capacity and endurance capability or perceive an increased fatigue [17, 19, 50].

It is generally believed that oxidative stress can affect negatively athletic performance and recovery [1, 3, 4]. By recent evidence, redox biomarkers measured in blood adequately reflect tissue redox status [51]; thus, the blood increased oxidative stress associated with OC use likely parallels increased free radicals also in muscle, implying potentially detrimental effects in sport performances [50].

Assessing the pro- and antioxidative stress effects of sport activity in the young female population may be complicated by several confounding factors [3], among these OC treatments can constitute a major confounder as shown by our present findings.

Elevation of oxidative stress implies several potential adverse effects including chronic diseases comprising cardiovascular disease (CVD), endothelial damage, thromboembolic events, and cancer [2, 52]. Notably, upper extremity thromboembolism in athletes [53], the so-called effort thrombosis, has been associated with use of hormonal contraception [54]. Whether increased risk of thromboembolism in OC-user female athletes is mediated by the increased oxidative stress needs further investigations.

There are limitations in our study. We studied young adult white female athletes, and thus, we cannot generalize these results to older athletes or sportswomen with different ethnic backgrounds; sport activities of athletes were heterogeneous ranging from aerobic to mixed aerobic-anaerobic and to anaerobic activity, and from elite to non-elite competing level, however, we recently showed that OC effects on high-sensitivity C-reactive protein (hsCRP), a biomarker of inflammation, did not vary according to the sport discipline practiced by female athletes [18]. We selected women taking monophasic combined (i.e., containing an estrogen plus a progestin) contraceptive pills, and we excluded other kinds of hormonal contraception; OCs were heterogeneous in type and amount of hormonal components although the majorities were OCs of third generation [18, 41]. We did not have detailed data about composition and dosing of potentially antioxidant supplements like vitamin E, C, and beta-carotene [25]. The observational nature of our study makes it impossible to determine if OC plays a causal role in the pathogenesis of elevated oxidative stress; however, a study by other authors seems to indicate a causative role [24]. In our study, the confidence intervals were somewhat large; however, highly significant results were obtained also after multivariate adjustments including several confounders. Finally, we evaluated oxidative stress by an assay measuring hydroperoxides (expression mainly of lipid peroxidation) [2, 24, 30], which constitutes only one of the possible indirect markers to assess oxidative stress status [10]; however, the FORT and FORD assays have been validated for clinical oxidative stress evaluation [32].

Strengths of the present study include assessment of oxidative stress and several lifestyles and alimentary habits, the homogeneous ethnic group, the rather narrow age range of competitive athletes, and strictly healthy subject inclusion.

Further studies have to be carried out to expand our observations including larger numbers of athlete of different sport disciplines and to better assess biochemical pathways related to oxidative stress elevation, the exact time-course of oxidative stress elevation according to OC use, the clinical significance, and the impact on athletic performance of this occurrence.

## Conclusions

Oxidative stress in competitive athletes is under study, but gender differences still need to be investigated. This was the first study (to our knowledge) demonstrating that OC use markedly elevates oxidative stress levels (according to hydroperoxide increase) in female athletes of different sport disciplines. We also investigated lifestyle and alimentary habits of study sportswomen. Conceivably, elevation of basal oxidative stress could favor increased concentrations of free radicals in the event of any pro-oxidative condition experienced by female athletes and may have implications for cardiovascular disease development, including “effort” thrombosis. Our findings highlighted that OC use should be considered by clinicians, coaches, sport trainers, and sportswomen themselves when developing strategies to reduce oxidative stress in female athletes.

## Abbreviations

BMI: Body mass index
CVD: Cardiovascular disease
FORD: Free oxygen radical defense
FORT: Free oxygen radical test
IQR: Interquartile
LDL: Low-density lipoprotein
NOS: Nitrogen oxigen species
OC: Combined oral contraceptive
RONS: Reactive oxigen nitrogen species
ROOH: Hydroperoxide
ROS: Reactive oxygen species
SD: Standard deviation
